# RAGE signaling in acute myocardial infarction: sRAGE as a dynamic biomarker and emerging therapeutic target

**DOI:** 10.3389/fendo.2026.1930897

**Published:** 2026-09-14

**Authors:** Aneta Aleksova, Elena Vianello, Paola Primignani, Massimiliano M. Corsi Romanelli, Lucia La Sala, Antonio P. Beltrami, Francesca Fumagalli, Elena Dozio

**Affiliations:** 1 Dipartimento Cardiotoracovascolare, Azienda Sanitaria Universitaria Giuliano Isontina, Trieste, Italy; 2 Dipartimento Universitario Clinico di Scienze Mediche Chirurgiche e della Salute, Università degli Studi di Trieste, Trieste, Italy; 3 Dipartimento di Scienze Biomediche per la Salute, Università degli Studi di Milano, Milan, Italy; 4 Laboratorio Sperimentale Ricerche Biomarcatori di Danno d’Organo, Istituto Auxologico Italiano IRCCS, Milan, Italy; 5 Dipartimento di Patologia Clinica e Sperimentale, Istituto Auxologico Italiano IRCCS, Milan, Italy; 6 IRCCS Multimedica, Milan, Italy; 7 Dipartimento di Medicina, Università degli Studi di Udine, Udine, Italy; 8 Istituto di Patologia Clinica, Azienda Sanitaria Universitaria Friuli Centrale, Udine, Italy; 9 Dipartimento di Danno Cerebrale e Cardiovascolare Acuto, Istituto di Ricerche Farmacologiche Mario Negri IRCCS, Milan, Italy

**Keywords:** biomarker, cardiac remodeling, inflammation, MACE, mortality, myocardial infarction, RAGE, sRAGE

## Abstract

The receptor for advanced glycation end products (RAGE) is a multiligand pattern-recognition receptor involved in inflammation, oxidative stress, and cardiometabolic disease. In acute myocardial infarction (AMI), activation of the AGE–RAGE signaling axis contributes to ischemia–reperfusion injury and post-infarction cardiac remodeling. Its soluble isoform (sRAGE) has emerged as a potential circulating biomarker related to RAGE pathway activity. Experimental studies support a role for RAGE in amplifying myocardial injury through inflammatory and oxidative mechanisms. In clinical settings, circulating sRAGE levels have been associated with markers of myocardial damage and disease severity; however, findings remain inconsistent, with both direct and inverse associations reported. Interpretation is further complicated by differences in sRAGE isoforms, heterogeneity in study design, timing of measurement, and lack of standardized analytical methods. Consequently, no validated cut-off values or clinical applications have been established. Limited longitudinal evidence suggests that the clinical significance of sRAGE may vary over the course of AMI. Higher acute-phase levels have been associated with myocardial injury and adverse outcomes in some cohorts, whereas longitudinal changes may reflect distinct biological processes and carry different prognostic information. These observations support further investigation of sRAGE kinetics as a potential complement to single time-point measurements, although prospective longitudinal validation is required before clinical implementation.

## Introduction

Cardiovascular diseases remain the leading cause of mortality worldwide, with acute myocardial infarction (AMI) representing a major contributor to both short- and long-term morbidity and mortality. Despite substantial advances in reperfusion strategies, including primary percutaneous coronary intervention and thrombolysis, myocardial ischemia–reperfusion (I/R) injury continues to be a major determinant of infarct size and subsequent cardiac remodeling, ultimately influencing the development of heart failure and adverse clinical outcomes (1). These processes are driven by a complex interplay of oxidative stress, inflammation, mitochondrial dysfunction, and regulated cell death.

Among the molecular pathways linking metabolic stress and inflammation, the receptor for advanced glycation end products (RAGE) has emerged as a key mediator of cardiovascular injury. RAGE is a multiligand pattern-recognition receptor encoded by the AGER gene that interacts with advanced glycation end products (AGEs) as well as endogenous damage-associated molecular patterns (DAMPs), including high mobility group box 1 (HMGB1) and members of the S100 protein family. Ligand binding activates intracellular signaling pathways that amplify oxidative stress and inflammatory responses, thereby contributing to myocardial injury and tissue remodeling (2–5).

Experimental evidence has consistently implicated the RAGE pathway in both the acute phase of myocardial I/R injury and the subsequent remodeling process. However, translating these mechanistic findings into clinically useful tools remains challenging. In particular, the extent to which activation of the RAGE pathway reflects disease severity or predicts clinical outcomes in patients with AMI has not yet been fully established.

Growing interest has therefore focused on soluble RAGE (sRAGE), a circulating isoform generated by alternative splicing or proteolytic cleavage of membrane-bound RAGE. Beyond acting as a decoy receptor, sRAGE has emerged as a potential biomarker related to the RAGE axis, with reported associations with myocardial injury, inflammatory burden, ventricular dysfunction, and cardiovascular outcomes. Nevertheless, published studies have produced apparently conflicting results, suggesting that the biological and clinical significance of circulating sRAGE may depend on the timing of measurement and the phase of myocardial injury.

In this Mini Review, rather than revisiting sRAGE as a prognostic biomarker per se, we focus on the less explored clinical relevance of its temporal kinetics following AMI. We critically examine the limited longitudinal evidence suggesting that the interpretation of circulating sRAGE may vary according to sampling time and phase of myocardial injury. We therefore propose a hypothesis-generating framework for future studies aimed at determining whether sRAGE trajectories provide information beyond single measurements, while highlighting the biological, analytical, and clinical factors currently limiting this interpretation.

### Literature search strategy

PubMed was searched through March 2026 using combinations of the terms “RAGE”, “sRAGE”, “acute myocardial infarction”, “acute coronary syndrome”, and “myocardial ischemia–reperfusion injury”, without restrictions on publication date or study design. Relevant clinical and preclinical studies were included, and reference lists of relevant reviews were screened for additional publications. No formal quality assessment was performed, consistent with the narrative nature of this Mini Review.

### Biological basis of RAGE signaling in AMI

RAGE is a multiligand pattern-recognition receptor belonging to the immunoglobulin superfamily that functions as a key regulator of sterile inflammation. Although constitutively expressed at low levels in most adult tissues, RAGE expression is markedly upregulated in response to oxidative stress, inflammation, and tissue injury, particularly in cardiomyocytes, endothelial cells, fibroblasts, and immune cells (2, 6). This inducible expression pattern distinguishes RAGE from many other pattern-recognition receptors and enables sustained amplification of inflammatory responses.

A unique feature of RAGE is its ability to recognize structurally different ligands. In the cardiovascular system, the most relevant include AGEs, HMGB1 and members of the S100 protein family. AGEs accumulate under conditions of oxidative stress, aging, diabetes, and metabolic dysfunction, whereas HMGB1 and S100 proteins are released by injured or activated cells following tissue damage, acting as DAMPs (2, 7–11).

Binding of these ligands to membrane-bound RAGE activates several intracellular signaling pathways, including MAPK, NADPH oxidase, and NF-κB. These pathways stimulate the production of reactive oxygen species, pro-inflammatory cytokines, chemokines, and adhesion molecules, while simultaneously increasing RAGE expression itself, thereby establishing a positive feedback loop that perpetuates inflammatory activation (6, 8). Consequently, RAGE functions not only as a sensor of tissue injury but also as an amplifier of chronic inflammatory responses.

In the setting of AMI, massive release of DAMPs from necrotic cardiomyocytes rapidly activates the RAGE pathway, linking oxidative stress to innate immune activation and subsequent ventricular remodeling. These biological properties provide the mechanistic basis for the involvement of RAGE throughout the different phases of myocardial injury and support the investigation of sRAGE as a circulating biomarker related to RAGE-axis biology.

### RAGE during myocardial injury

Activation of the RAGE pathway represents one of the earliest molecular events following I/R injury. Experimental studies have consistently demonstrated that RAGE expression rapidly increases within the ischemic myocardium and that both genetic deletion and pharmacological inhibition of the receptor significantly reduce infarct size and improve functional recovery after reperfusion (12–15). These observations identify RAGE as an active mediator rather than a simple marker of tissue injury.

The detrimental effects of RAGE activation arise from the convergence of multiple interconnected mechanisms. Engagement of membrane-bound RAGE by AGEs and endogenous DAMPs enhances oxidative stress through activation of NADPH oxidase and mitochondrial dysfunction, while simultaneously activating NF-κB-dependent inflammatory signaling. This results in increased production of pro-inflammatory cytokines, including TNF-α, IL-1β, and IL-6, together with upregulation of adhesion molecules that promote leukocyte recruitment and sustain myocardial inflammation (2, 14). In parallel, increased inducible nitric oxide synthase expression contributes to nitrosative stress and further compromises cardiomyocyte survival (14).

Among RAGE ligands, HMGB1 appears to play a particularly important role during the acute phase of AMI. Released from necrotic cardiomyocytes and activated immune cells, HMGB1 amplifies inflammatory signaling through interaction with RAGE, thereby extending tissue injury beyond the initial ischemic insult. Consistent with this mechanism, experimental inhibition of the HMGB1–RAGE pathway reduces infarct size and improves cardiac function following I/R injury (3, 16).

The impact of RAGE signaling is not confined to the acute phase. Persistent activation of the RAGE pathway contributes to adverse ventricular remodeling by promoting fibroblast activation, extracellular matrix deposition, myocardial fibrosis, and progressive ventricular dysfunction. Together with sustained oxidative stress and chronic inflammation, these mechanisms facilitate the transition from acute myocardial infarction to chronic heart failure (14–18).

Taken together, experimental evidence supports a dual role for RAGE throughout the natural history of AMI. During the acute phase, RAGE amplifies ischemia–reperfusion injury, whereas persistent pathway activation promotes maladaptive ventricular remodeling. This temporal involvement provides the biological rationale for interpreting circulating sRAGE as a biomarker whose significance may evolve throughout the different stages of myocardial injury.

### sRAGE as a biomarker

sRAGE has attracted considerable attention as a circulating biomarker related to the RAGE axis, as it may reflect inflammatory processes involved in myocardial injury. By lacking the transmembrane and intracellular domains, sRAGE acts as a soluble decoy receptor that binds RAGE ligands, including AGEs, HMGB1, and S100 proteins, thereby limiting membrane-bound RAGE activation and downstream inflammatory signaling (6).

However, circulating sRAGE levels result from the interplay of multiple processes, including alternative splicing, membrane RAGE shedding, ligand interactions, and clearance. Therefore, sRAGE concentrations should not be interpreted as a direct surrogate of tissue RAGE activation.

sRAGE comprises two biologically distinct isoforms. Endogenous secretory RAGE (esRAGE) is generated by alternative splicing of the *AGER* gene and is directly released into the circulation (19), whereas cleaved RAGE (cRAGE) is produced by proteolytic shedding of membrane-bound RAGE, primarily mediated by ADAM10 and MMP-9 (20, 21). Because of their distinct biological origins, these isoforms may also have different biological and prognostic significance, with cRAGE more closely reflecting membrane RAGE expression and shedding activity.

Importantly, most clinical studies have measured total sRAGE using non-standardized assays and have not measured cRAGE and esRAGE separately. This methodological limitation represents a major source of heterogeneity and may partly explain the inconsistent clinical and prognostic associations reported across studies. Furthermore, separately evaluating the concentrations of the different sRAGE isoforms could improve the prognostic performance of this biomarker.

### sRAGE during the acute phase of AMI

Several clinical studies have reported increased circulating sRAGE during the acute phase of AMI and associations with markers of myocardial injury or disease severity, including cardiac troponin release, impaired left ventricular function, and infarct size. However, these associations have not been consistent across all populations (22–24). Higher baseline sRAGE levels have also been associated with an increased risk of in-hospital complications and major adverse cardiovascular events (MACE), independently of established biomarkers and clinical risk scores (24, 25). These findings suggest that elevated acute-phase sRAGE may be associated with the magnitude of myocardial injury and inflammatory activation in selected clinical settings (**Table 1**).

**Table 1:** Clinical studies evaluating the temporal and prognostic significance of circulating sRAGE in AMI.

Population	Study design	Timing	Follow-up	Main findings	Prognostic implications	Key sRAGE values
ACS n=215 (47.4% STEMI) (25)	Prospective observational cohort	Admission	Median 366 days *(IQR 273–519)*	Acute sRAGE ↑ associated with in-hospital events (cardiac death, reinfarction, new-onset heart failure)	Early risk stratification	Mean 1045 ± 850 pg/mL; ROC-derived cut-off ≈1094 pg/mL for predicting in-hospital adverse events
ACS n=524 (39% STEMI) (24)	Prospective observational cohort	Admission (≤24h) + 6 weeks (n=114)	Median 25.7 months *(IQR 13.3–38.3)*	Acute sRAGE ↑ predicts MACE; increase in sRAGE from baseline to 6 weeks → lower risk of recurrent ACS	Phase-dependent prognostic value	Acute median 28.4 au *(IQR: 21.6-37.5)* 6-w median 32.6 au *(IQR: 26.7-41.0)*
67 consecutive patients with *de novo* STEMI treated with primary PCI (28)	Prospective observational pilot study	Peripheral blood sampling within 24 h after STEMI (post-PCI)	Baseline echocardiography (≤48 h) and 6-month follow-up	Higher sRAGE associated with lower baseline LVEF and inversely associated with left atrial remodeling, but not with LV remodeling	Marker of baseline cardiac dysfunction and left atrial remodeling rather than LV remodeling	Baseline median 856.6 pg/mL (IQR 527.0–1355.5); no prognostic cut-off identified.
80 STEMI patients treated with primary PCI (23)	Prospective observational cohort	Before PCI, immediately after PCI, and on days 1 and 2 after PCI	Echocardiography at baseline (1–3 days) and at a median of 7 months; CMR at 1–3 days in a subgroup (n = 34)	Dynamic increase after reperfusion, peaking immediately after PCI before troponin I and declining within 48 h; peak levels predicted lower LVEF and larger infarct size	Dynamic biomarker of myocardial injury and predictor of long-term cardiac dysfunction	Before PCI: 3804 ng/L (IQR 2522–5281); immediately after PCI: 4535 ng/L (3155–6129); day 1: 1446 ng/L (1015–2741); day 2: 1371 ng/L (902–3121); no prognostic cut-off identified.

Evidence from clinical studies across acute and chronic settings on the prognostic value of sRAGE measured using assays that did not distinguish between cRAGE and esRAGE. Reported sRAGE concentrations are presented in the original units used by each study. Only studies reporting longitudinal clinical outcomes were included. Studies using proximity extension assay (PEA) report sRAGE as arbitrary units (au), whereas studies using immunoassays report concentrations in pg/mL. ACS, acute coronay syndrome; CAD, coronay artery disease; ROC, Receiver Operating Characteristic curve; IQR, Interquartile Range; SA, stable angina; UA, unstable angina; LVEF, left ventricular ejection fraction; PCI, Percutaneous Coronary Intervention; CRM, cardiac magnetic resonance.

↑ means increased level; → means is associated with.

### The importance of timing

Despite these associations, the clinical interpretation of circulating sRAGE remains controversial. Several studies have reported inverse associations between sRAGE concentrations and disease severity, particularly in patients with NSTEMI, while lower circulating levels have also been observed in AMI compared with stable coronary disease (26, 27). These apparently discordant observations may partly reflect differences in the timing of measurement, although differences in patient phenotype, renal function, reperfusion, assay methodology and sRAGE isoforms may also contribute.

The limited longitudinal evidence available raises the possibility that sRAGE may behave as a dynamic biomarker (**Table 1**). While higher concentrations during the acute phase have been associated with greater myocardial damage and poorer short-term outcomes in some cohorts, changes occurring during recovery have been hypothesized to reflect compensatory ligand-scavenging or inflammatory-modulating mechanisms (24). However, this interpretation remains speculative and requires mechanistic validation.

In agreement with this concept, increases in circulating sRAGE during follow-up have been associated with a lower risk of recurrent acute coronary events, whereas single baseline measurements incompletely capture these temporal changes (24, 25). Similarly, sRAGE measured during the chronic phase has been associated with left ventricular function and atrial remodeling, although its ability to predict adverse ventricular remodeling remains uncertain (28).

### Why are current data heterogeneous?

Current clinical evidence has important limitations. Most studies are observational, frequently single-center, and include heterogeneous ACS populations, with sample sizes ranging from fewer than 100 to approximately 500 participants. To date, only a few long-term prospective studies have evaluated the prognostic significance of sRAGE. Only a minority performed serial sRAGE measurements, limiting evaluation of its temporal trajectory. Differences in STEMI/NSTEMI prevalence, sampling time, follow-up duration, endpoints, and assay methods further hinder comparisons. Moreover, most studies measured total sRAGE without distinguishing between cleaved RAGE (cRAGE) and endogenous secretory RAGE (esRAGE), despite their distinct biological origins and potentially different clinical significance. Age, diabetes, renal function, inflammatory burden, and concomitant therapies may influence sRAGE concentrations and were not uniformly controlled across studies. Kidney dysfunction deserves particular consideration because altered renal handling may influence circulating sRAGE concentrations independently of the severity of myocardial injury. Analytical and preanalytical variability, including assay platform, antibody specificity, calibration, sample handling and reporting units, represents an additional source of between-study heterogeneity. Consequently, the available evidence does not yet establish the incremental prognostic value of sRAGE, supporting the need for larger, multicenter prospective studies with standardized serial measurements and appropriate adjustment for potential confounders.

### Clinical implications

At present, sRAGE should be regarded as an investigational biomarker that may provide complementary information related to RAGE-axis biology and myocardial injury during AMI. Its clinical application remains limited by the lack of validated cut-offs and evidence demonstrating incremental prognostic value beyond cardiac troponins, natriuretic peptides, inflammatory markers, and validated clinical risk models. Future studies should therefore determine whether serial sRAGE assessment and its integration into multimarker strategies provide clinically meaningful information beyond conventional approaches.

In some cohorts, higher acute-phase sRAGE concentrations have been associated with larger infarct size, impaired ventricular function, and adverse cardiovascular events. Limited longitudinal evidence further suggests that changes in circulating sRAGE during recovery may carry additional prognostic information, although their biological interpretation and incremental clinical value remain to be established (24, 25).

Despite these promising findings, routine clinical implementation remains premature because of the lack of standardized analytical methods, validated reference ranges, and assays capable of distinguishing cRAGE from esRAGE. Although commercial immunoassays are available for the measurement of esRAGE, cRAGE is generally estimated by subtracting esRAGE from total sRAGE concentrations, an indirect approach that may introduce additional analytical variability and potential measurement error. Moreover, circulating sRAGE concentrations are influenced by several clinical variables, including age, diabetes, renal function, and concomitant therapies, complicating interpretation of isolated measurements (29–31).

Rather than serving as a standalone biomarker, sRAGE may ultimately find application within multimarker strategies. Its potential clinical value may depend not only on absolute concentrations but also on temporal trajectories, which could provide complementary information on disease evolution and recovery.

### Therapeutic perspectives

Therapeutic targeting of the RAGE pathway remains mechanistically promising but clinically unvalidated. Experimental studies have consistently shown that genetic deletion of RAGE, administration of exogenous sRAGE, HMGB1 neutralization, and direct RAGE inhibition reduce myocardial injury, attenuate adverse ventricular remodeling, and improve cardiac function following ischemia–reperfusion injury. However, these findings derive predominantly from *in vitro* and animal studies and cannot yet be translated into therapeutic recommendations for patients with AMI.

Among the proposed approaches, exogenous sRAGE has been extensively investigated as a decoy strategy. Experimental administration of sRAGE or genetic deletion of RAGE reduces infarct size, inflammatory responses, and functional impairment following myocardial I/R injury (14, 15). More recent evidence suggests that sRAGE may also modulate immune responses by reducing IL-6 production and promoting regulatory T-cell differentiation through the JAK2/STAT3–PD-L1 pathway (32, 33).

Additional therapeutic strategies target other components of the RAGE signaling network. HMGB1 neutralization has shown protective effects in experimental models by reducing inflammatory cell infiltration and limiting myocardial injury, supporting the HMGB1–RAGE pathway as a promising therapeutic target (34, 35). Similarly, direct inhibition of RAGE through neutralizing antibodies, small-molecule inhibitors, or gene-silencing approaches has demonstrated beneficial effects on inflammation, apoptosis, infarct size, and ventricular remodeling in preclinical studies (12, 16, 36, 37).

Interestingly, modulation of the RAGE pathway may also contribute to the cardioprotective effects of therapies already used in clinical practice. Experimental evidence suggests that statins and inhibitors of the renin–angiotensin system can reduce RAGE activation by decreasing ligand burden and modulating receptor expression (38, 39). Although these observations remain largely mechanistic, they suggest that RAGE signaling may already be partially modulated by conventional cardiovascular therapies.

Several pharmacological challenges currently limit the clinical translation. Preclinical studies indicate that exogenous sRAGE is rapidly cleared following administration, representing a potential pharmacokinetic limitation for therapeutic application (40). Moreover, as a broad decoy receptor, sRAGE may non-selectively sequester multiple ligands, potentially interfering with physiological RAGE-dependent signaling beyond the injured myocardium. Alternative strategies, including small-molecule RAGE antagonists or modulation of RAGE shedding, may offer greater pharmacological feasibility, although their efficacy and safety remain to be established. (6, 41, 42).

Future translational studies should first establish safety, pharmacokinetics, target engagement, and optimal timing of intervention before evaluating whether modulation of the AGE–RAGE pathway can improve clinical outcomes beyond current guideline-directed therapies in patients with AMI.

### Future directions

Although substantial progress has been made in understanding the role of the RAGE pathway in myocardial injury, several challenges must be addressed before sRAGE can be incorporated into routine clinical practice.

First, future studies should move beyond single time-point measurements and adopt longitudinal sampling strategies. Limited evidence suggests that the biological and prognostic significance of circulating sRAGE may vary throughout the course of AMI, supporting further investigation of whether temporal profiles provide information beyond isolated measurements.

Second, analytical standardization remains a major priority. Harmonization of assay methodologies and implementation of isoform-specific measurements capable of distinguishing cRAGE from esRAGE will be essential to improve comparability among studies and facilitate clinical interpretation.

Third, larger prospective multicenter cohorts are required to validate the incremental prognostic value of sRAGE beyond established biomarkers such as cardiac troponins, natriuretic peptides, and inflammatory markers. Rather than replacing existing biomarkers, sRAGE should be evaluated as part of integrated multimarker strategies reflecting complementary biological processes.

Another important priority will be the establishment of standardized sampling protocols. Because circulating sRAGE concentrations appear to change rapidly during the acute and recovery phases of AMI, defining the optimal timing for blood collection may prove as important as analytical standardization itself. Future longitudinal studies should therefore evaluate serial measurements spanning hospital admission and reperfusion, early recovery, and post-discharge follow-up, using longitudinal modeling to identify reproducible sRAGE trajectory patterns. These trajectories should then be externally validated and evaluated in relation to infarct healing, ventricular remodeling, long-term cardiovascular outcomes, and their incremental prognostic value beyond established biomarkers and clinical risk models.

Finally, the growing understanding of the RAGE pathway opens new opportunities for precision cardiovascular medicine. Combining biomarker profiling with targeted modulation of RAGE signaling may enable identification of patient subgroups who could benefit from personalized therapeutic approaches. Whether dynamic monitoring of sRAGE can guide treatment decisions or identify patients most likely to respond to RAGE-targeted interventions remains an important objective for future translational research.

## Conclusions

The AGE–RAGE signaling pathway plays a central role in the pathophysiology AMI by linking oxidative stress, inflammation, and adverse ventricular remodeling (**Figure 1**). Experimental evidence consistently supports its contribution to myocardial ischemia–reperfusion injury, while clinical studies identify soluble RAGE (sRAGE) as a potential circulating biomarker related to RAGE-axis biology and myocardial injury.

**Figure 1:**
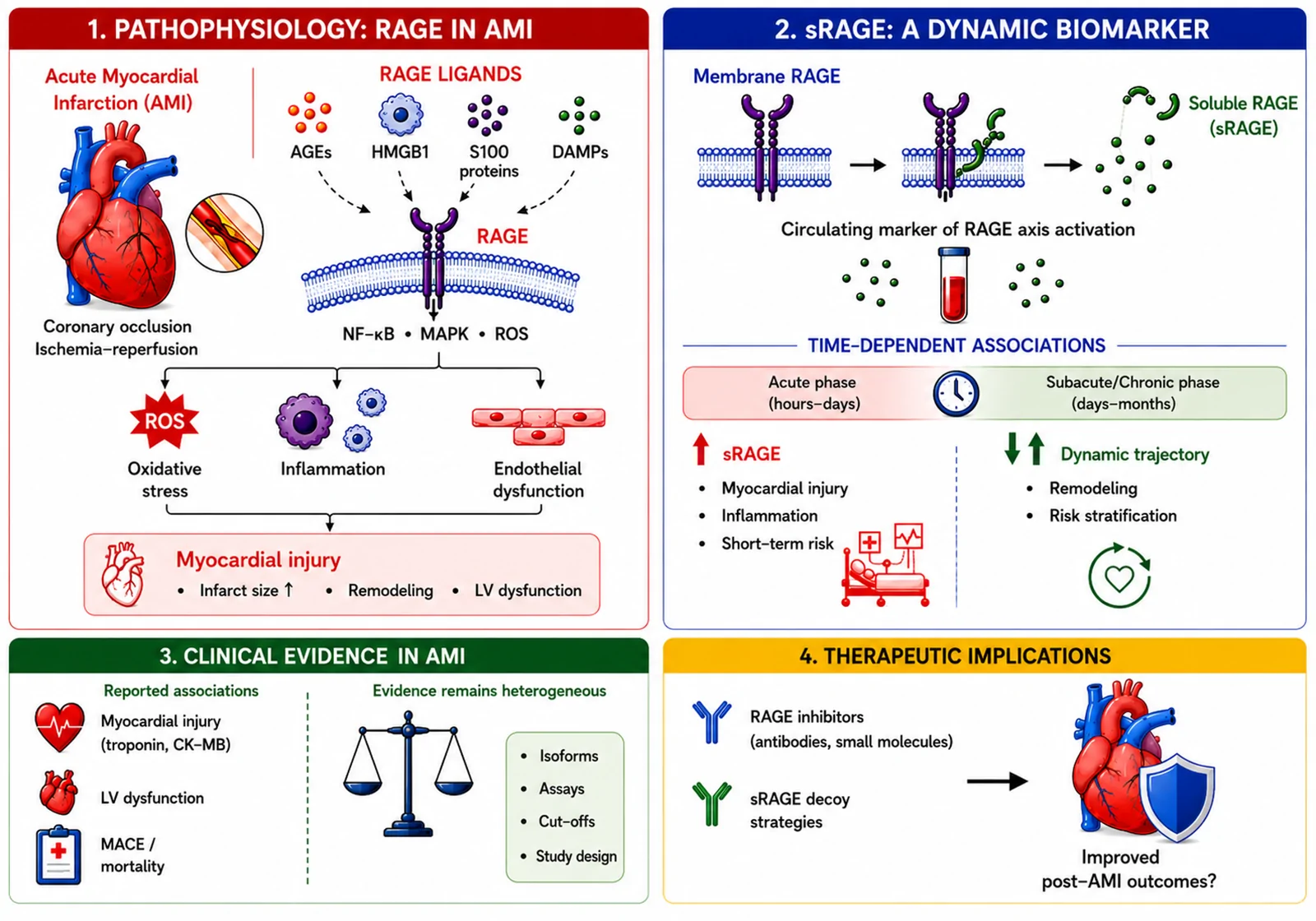
Graphical overview of RAGE signaling across the different phases of acute myocardial infarction. Activation of membrane-bound RAGE by AGEs, HMGB1, S100 proteins, and other DAMPs promotes oxidative stress, inflammation, and myocardial remodeling following AMI. Soluble RAGE (sRAGE) is a circulating biomarker related to the RAGE axis whose clinical interpretation may vary according to the timing and phase of myocardial injury. Current clinical evidence remains heterogeneous, highlighting the need for standardized longitudinal studies to validate its role in risk stratification and therapeutic monitoring.

Available clinical studies suggest that the interpretation of circulating sRAGE may vary according to the timing of measurement and phase of myocardial injury. However, the limited longitudinal evidence and substantial methodological heterogeneity currently preclude the definition of consistent phase-specific associations.

Current evidence raises the hypothesis that sRAGE may be more informative when interpreted dynamically rather than as an isolated measurement. However, the limited longitudinal evidence does not yet allow the definition of validated trajectory patterns or establish their incremental prognostic value beyond conventional biomarkers and clinical risk models. Standardized prospective studies incorporating serial and isoform-specific measurements are therefore needed to determine whether sRAGE kinetics can improve risk stratification and potentially inform therapeutic decision-making in patients with AMI.
